# Leptin induced GRP78 expression through the PI3K-mTOR pathway in neuronal cells

**DOI:** 10.1038/srep07096

**Published:** 2014-11-18

**Authors:** Mina Thon, Toru Hosoi, Michiko Yoshii, Koichiro Ozawa

**Affiliations:** 1 Department of Pharmacotherapy, Graduate School of Biomedical and Health Sciences, Hiroshima University, 1-2-3 Kasumi, Minami-ku, Hiroshima 734-8551, Japan

## Abstract

Leptin is a circulating hormone that plays a critical role in regulating energy expenditure and food intake. Evidence to suggest the involvement of endoplasmic reticulum (ER) stress in the development of obesity is increasing. To adapt against ER stress, cells trigger the unfolded protein response (UPR). The 78 kDa glucose-regulated protein (GRP78) is an ER chaperone that protects cells against ER stress by inducing protein folding. In the present study, we hypothesized that leptin may activate UPR and protect against ER stress associated with obesity. SH-SY5Y, a human neuroblastoma cell line stably transfected with the Ob-Rb leptin receptor (SH-SY5Y-ObRb), was treated with leptin. We demonstrated that leptin induced GRP78 expression. We then validated the mechanism responsible for the leptin-induced expression of GRP78. Interestingly, leptin-induced GRP78 expression was not dependent on IRE1-XBP1 pathway. On the other hand, the PI3K inhibitor, LY294002, and mTOR inhibitor, rapamycin, inhibited the leptin-induced expression of GRP78. These results suggested that the leptin-induced expression of GRP78 may be dependent on the PI3K-mTOR pathway. Leptin specifically induced GRP78 because the induction of the ER-apoptotic marker, CHOP, was not detected in leptin-treated cells. Therefore, leptin may upregulate the expression of GRP78, thereby protecting against ER stress associated with obesity.

Obesity is a serious health concern due to its high morbidity and mortality rates^1^. It is also a risk factor for several diseases including hyperlipidemia, hyperinsulinemia, hypertension, and atherosclerosis; however, the underlying mechanisms remain poorly understood^2^.

Leptin, a hormone secreted from adipocytes, acts centrally as a regulator of body weight by suppressing food intake and enhancing energy expenditure^3^. The cellular mechanisms of leptin are known to be initiated by binding to specific receptors (Ob-R) localized in the cell membrane^4^. Leptin signaling occurs predominantly through the Ob-Rb long isoform of the leptin receptor, following by activation of the JAK-STAT pathway. Leptin has also been shown to activate ERK and Akt, two key signal-transduction pathways associated with cell growth^5^. Due to its anti-obesity effects, leptin has attracted interest regarding treatments for obesity. However, leptin resistance, as signified by high circulating levels of leptin^6^, represents a major problem in the treatment of obese patients. The mechanism underlying leptin resistance has yet to be elucidated in detail.

The endoplasmic reticulum (ER) is an organelle that plays important roles in multiple cellular processes including the regulation of intracellular calcium homeostasis, biosynthesis of steroids, protein folding, transport in secretory pathways, and quality control of newly made proteins^7^,^8^. Multiple disturbances including hypoxia, energy deprivation, perturbations in calcium homeostasis, alterations in the oxidation–reduction balance, hyperhomocysteinemia, metabolic demands, and viral infections have been shown to disrupt ER homeostasis. Cells trigger an evolutionarily-conserved response, termed the unfolded protein response (UPR), to adapt to stress originating from the ER^9^,^10^. UPR is a feedback mechanism that prevents the accumulation of misfolded or unfolded proteins in the lumen of the ER. The UPR also initiates adaptive responses to restore normal cell function, including the inhibition of general protein translation and an increase in the production of chaperone proteins, such as glucose-regulated protein 78 (GRP78)^11^,^12^,^13^. GRP78 is a molecular chaperone that is located in the lumen of the ER and its expression is known to be induced during ER stress. GRP78 binds newly synthesized proteins as they are translocated into the ER, and maintains them in a state competent for subsequent folding and oligomerization. It also controls the activation of the transmembrane ER stress sensors, PKR-like endoplasmic reticulum-resident kinase (PERK), activating transcription factor 6 (ATF6), and inositol-requiring enzyme α (IRE1α) through binding-release mechanisms^14^. GRP78 acts as an anti-apoptotic regulator by protecting cells against ER stress-induced cell death^15^,^16^. A previous study demonstrated that prolonged ER stress activated apoptotic signals and caused cell death^17^,^18^,^19^,^20^.

ER stress has been associated with the development of neurodegenerative diseases, cancer, diabetes, and obesity. Current evidence suggests that overnutrition may contribute to the development of ER stress and activation of the UPR signaling pathway^21^,^22^. In accordance with this finding, we and others have reported the involvement of ER stress in leptin resistance, which is one of the mechanisms responsible for the pathogenesis of obesity^22^,^23^,^24^. Nevertheless, the mechanisms underlying ER stress and activation of UPR signaling in obesity have yet to be elucidated in detail. In the present study, we hypothesized that leptin may be able to activate the UPR in neuronal cells.

## Results

### Leptin induced the phosphorylation of STAT3, Akt, and ERK in SH-SY5Y-ObRb cells

**Figure 1:**
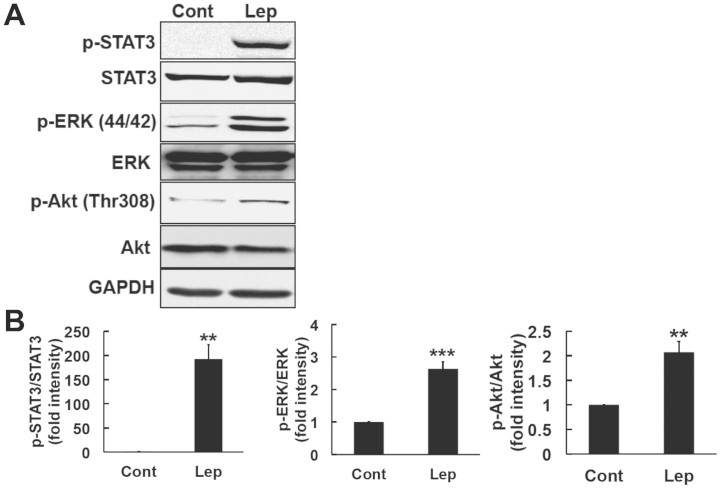
Leptin activated STAT3, ERK, and Akt in SH-SY5Y-ObRb cells. (A). SH-SY5Y-ObRb cells were treated with leptin 0.5 μg/ml for 30 min, and the levels of phospho-STAT3 (Tyr705), STAT3, phospho-ERK1/2 (Thr202/Tyr204), ERK, phospho-Akt (Thr308), Akt, and GAPDH were then analyzed by Western blotting. Leptin increased the phosphorylation of STAT3 (Tyr705), ERK (Thr202/Tyr204), and Akt (Thr308). (B). Densitometric analysis of phospho-STAT3 (Tyr705), phospho-ERK1/2 (Thr202/Tyr204), and phospho-Akt (Thr308) levels using image analyzing software. Data are expressed as the mean ± S.E. of 3 independent experiments (n = 3). ** *P* < 0.01; *** *P* < 0.001.

Leptin has been shown to activate the JAK2-STAT3 signaling pathway through the Ob-Rb long isoform of the leptin receptor^25^,^26^,^27^. Previous studies have also shown that leptin can induce the phosphorylation of Akt and ERK^28^,^29^. In the present study, we used the SH-SY5Y human neuroblastoma cell line stably transfected with the Ob-Rb leptin receptor (SH-SY5Y-ObRb)^30^. Cells were treated with leptin (0.5 μg/ml, 30 min) and the activation of STAT3, Akt, and ERK was analyzed by Western blotting. As shown in Figure 1, leptin markedly increased the phosphorylation of STAT3, Akt, and ERK in the SH-SY5Y-ObRb cell line. These results indicated that the leptin signal was functionally activated under the present experimental conditions (Fig. 1A and B).

### Leptin induced GRP78 expression in SH-SY5Y-ObRb cells

ER stress was previously shown to upregulate the expression of GRP78. Moreover, GRP78 plays protective roles against ER stress by promoting folding to prevent the aggregation of proteins^31^.

**Figure 2:**
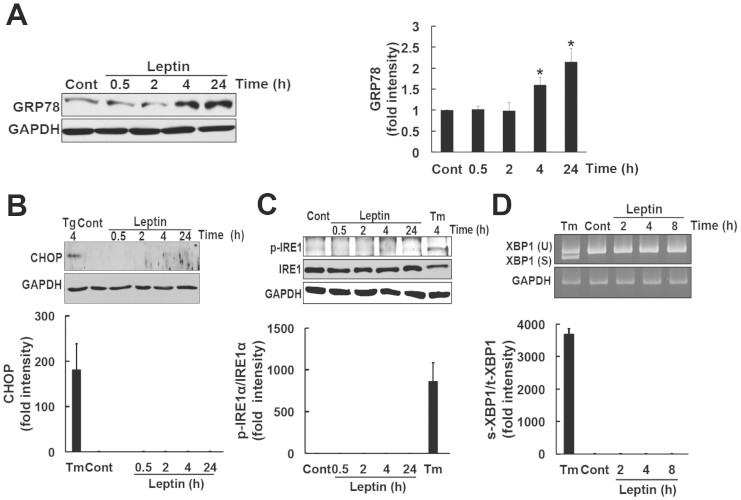
Leptin induced GRP78, but not CHOP or IRE1 phosphorylation in SH-SY5Y-ObRb cells. (A). SH-SY5Y-ObRb cells were treated with leptin (0.5 μg/ml) for 0.5, 2, 4, and 24 h. Leptin significantly increased the expression of GRP78 after 4 and 24 h. Densitometric analysis of GRP78 levels using image analyzing software. Data are expressed as the mean ± S.E. of 3 independent experiments (n = 3). * *P* < 0.05. (B). Cells were treated with thapsigargin (Tg) (0.2 μM, 4 h) as the positive control. Leptin did not induce the expression of CHOP. Densitometric analysis of CHOP using image analyzing software. Typical data of 3 independent experiments were shown. (C). Leptin did not induce the phosphorylation of IRE1α. Tunicamycin (Tm) (1 μg/ml, 4 h) was used as the positive control. Densitometric analysis of phospho-IRE1α using image analyzing software. Typical data of 3 independent experiments were shown. (D). Leptin did not induce a spliced form of XBP1. SH-SY5Y-ObRb cells were treated with leptin (0.5 μg/ml) for 2, 4, and 8 h. Tunicamycin (Tm) (1 μg/ml, 8 h) was used as the positive control. Densitometric analysis of XBP1 mRNA expression levels using image analyzing software. Typical data of 3 independent experiments were shown.

Using the SH-SY5Y-ObRb cell line, we determined whether leptin could induce the expression of GRP78. SH-SY5Y-ObRb cells were treated with leptin (0.5 μg/ml) for 0.5, 2, 4, and 24 h. Proteins were then isolated and analyzed by Western blotting. Leptin increased the expression of GRP78 after 4 and 24 h (Fig. 2A). We previously reported that ER stress contributes to leptin resistance^23^. Therefore, it would be interesting to analyze the effect of leptin-induced GRP78 in leptin-resistance model. Briefly, SH-SY5Y-ObRb cells were pretreated with Tm (0.05 μg/ml, 4 h) followed by leptin (0.5 μg/ml, 24 h). As shown in supplementary figure 4, pretreatment with Tm did not affect leptin-induced GRP78. Thus, GRP78 levels would not be increased in ER stressed model. We next examined the mechanism responsible for the leptin-induced expression of GRP78. The induction of GRP78 is known to be mediated through the inositol-requiring enzyme 1-X-box-binding protein 1 (IRE1-XBP1) pathway. IRE1 is an ER transmembrane sensor that activates the UPR to maintain ER and cellular functions. The activation of IRE1 induces the splicing of XBP1 mRNA. XBP1 translated from spliced mRNA has been shown to act as a transcriptional factor for UPR-regulated genes such as the GRP78 gene^32^,^33^. Therefore, we determined whether leptin could activate the phosphorylation of IRE1. Cells were treated with leptin (0.5 μg/ml) for 0.5, 2, 4, and 24 h, and Western blotting was then performed. The ER stress inducer that interferes with protein glycosylation; i.e., tunicamycin was used as a positive control. Unfortunately, leptin did not activate IRE1 at the times investigated (Fig. 2C). We simultaneously investigated whether leptin could activate XBP1 splicing. Cells were treated with leptin (0.5 μg/ml) for 2, 4, and 8 h, and XBP1 mRNA expression levels were analyzed by RT-PCR. Tm was used as a positive control. XBP1 splicing was not detected in leptin-treated cells (Fig. 2D). These results indicated that leptin-induced GRP78 was not mediated through the IRE1-XBP1 pathway.

SH-SY5Y-ObRb cells were treated with leptin (0.5 μg/ml) to determine whether leptin activated other UPR-regulated genes. UPR-related genes including, ATF6 and eukaryotic initiation factor 2α (eIF2α) were analyzed by Western blotting. Treating the cells with Tm increased ATF6 processing which resulted in reduced amount of the full-length form of ATF6 (Supplementary Fig. 1). However, leptin did not affect ATF6 levels (Supplementary Fig. 1). On the other hand, we found that leptin slightly induced the phosphorylation of eIF2α (Supplementary Fig. 2).

The ER elicits apoptotic signals when its function is severely impaired. One of the components of the ER stress-related apoptotic transcription factor is the C/EBP homologous protein (CHOP), also known as growth arrest- and DNA damage-inducible gene 153 (GADD153)^34^,^35^. Since we demonstrated that leptin activated UPR, as signified by the upregulation of GRP78; we subsequently investigated whether leptin could also activate CHOP. To obtain a positive control for the expression of CHOP, SH-SY5Y-ObRb cells were treated with the ER stress inducer thapsigargin (Tg). We successfully detected CHOP in SH-SY5Y-ObRb cells after a 4-h treatment with Tg (Fig. 2B). We then analyzed the expression of CHOP with the leptin treatment. Leptin did not induce the expression of CHOP (Fig. 2B). This result suggested that leptin may have specifically activated the GRP78 and eIF2α phosphorylation signaling pathways, and also that leptin-induced eIF2α phosphorylation may not be linked to the CHOP expression.

### Involvement of PI3K in the leptin-induced expression of GRP78 in SH-SY5Y-ObRb cells

**Figure 3:**
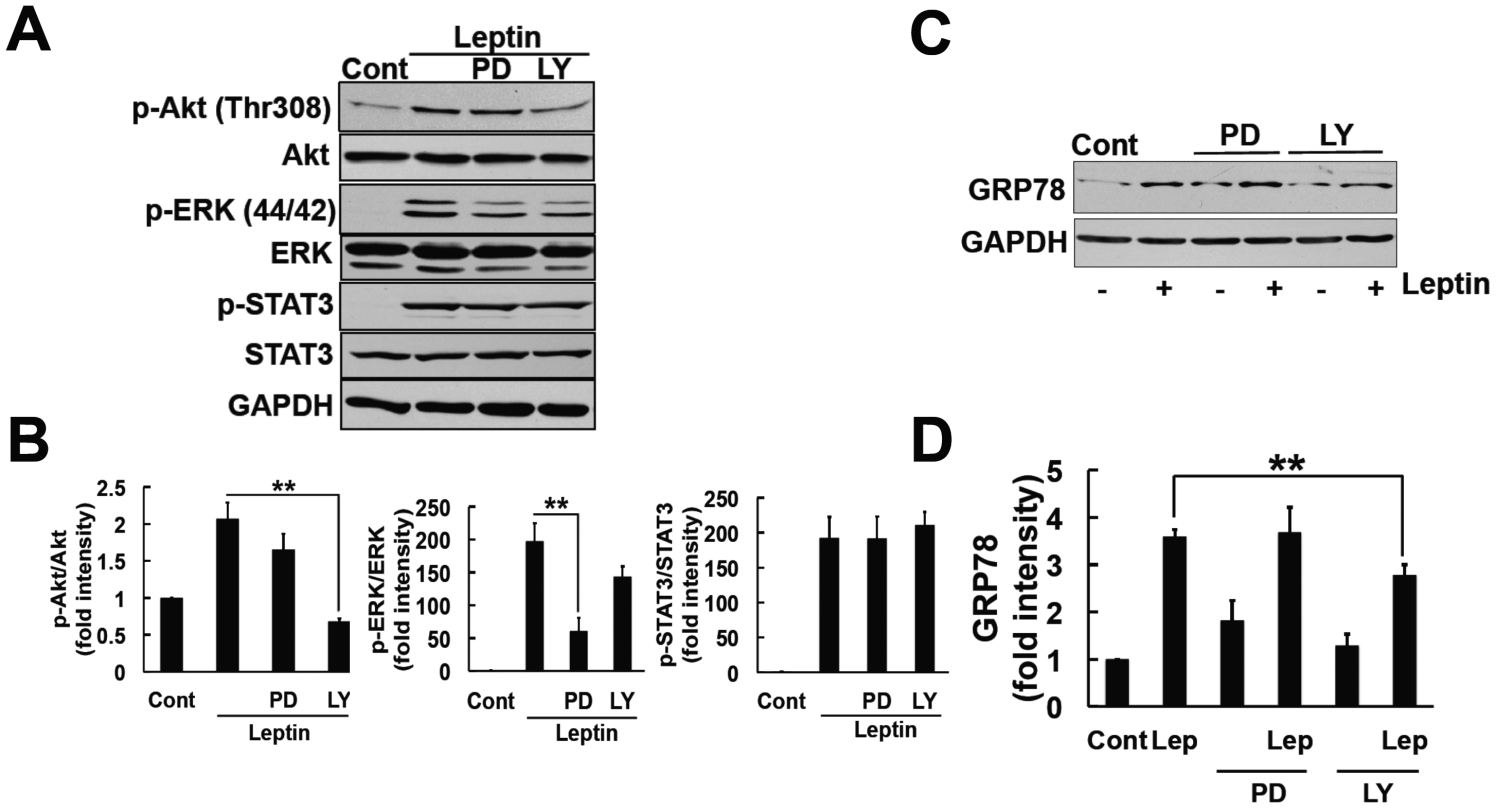
Involvement of PI3K in the leptin-induced expression of GRP78. (A). SH-SY5Y-ObRb cells were pretreated with PD98059 (PD; 10 μM) or LY294002 (LY; 5 μM) for 30 min followed by leptin (0.5 μg/ml, 30 min). Western blotting analysis was performed using specific antibodies for phospho-Akt (Thr308), Akt, phospho-ERK 1/2 (Thr202/Tyr204), ERK, phospho-STAT3 (Tyr705), STAT3, and GAPDH. (B). Densitometric analysis of phospho-Akt (Thr308), phospho-ERK1/2 (Thr202/Tyr204), and phospho-STAT3 (Tyr705) levels using image analyzing software. The treatment with PD inhibited the phosphorylation of ERK, but not Akt. The treatment with LY significantly inhibited the phosphorylation of Akt and also slightly inhibited that of ERK. The treatment with PD or LY did not inhibit the phosphorylation of STAT3. Data are expressed as the mean ± S.E. of 3 independent experiments (n = 3). ** *P* < 0.01. (C). SH-SY5Y-ObRb cells were pretreated with PD98059 (PD; 10 μM) or LY294002 (LY; 5 μM) for 30 min followed by leptin (0.5 μg/ml, 24 h). Western blotting analysis was performed using antibodies for GRP78 and GAPDH. (D). A densitometric analysis of GRP78 levels were performed using image analyzing software. LY hindered the effects of leptin on the induction of GRP78 expression. Data are expressed as the mean ± S.E. of 4 independent experiments (n = 4). ** *P* < 0.01.

The mechanism responsible for the leptin-induced expression of GRP78 has not yet been determined. We examined other signaling pathways activated by leptin. Consistent with previous findings^36^,^37^, we observed the activation of Akt and ERK in the SH-SY5Y-ObRb model (Fig. 1A and B). Based on these results, we investigated whether leptin-activated Akt and ERK mediated the expression of GRP78. We used specific inhibitors of PI3K and MEK, LY294002 and PD98059, respectively. To ensure the efficacy and specificity of each inhibitor, SH-SY5Y-ObRb cells were pretreated with LY294002 (5 μM) and PD98059 (10 μM) for 30 min, followed by leptin. After a 30-min stimulation with leptin, protein was isolated and analyzed using phospho-specific antibodies for Akt and ERK1/2. LY294002 significantly inhibited the phosphorylation of Akt. The slight inhibition of ERK phosphorylation was also observed by LY294002. This result was consistent with previous findings in which PI3K activity was responsible for activating the Raf/MEK1/ERK cascade^38^. On the other hand, PD98059 inhibited the phosphorylation of ERK, but not Akt (Fig. 3A and B). Therefore, LY294002 and PD98059 specifically inhibited the activation of PI3K and MEK. We also determined whether the leptin-mediated activation of STAT3 was dependent on Akt and ERK1/2 under the same conditions. The pretreatment of neuroblastoma cells with LY294002 and PD98059 did not alter the leptin-mediated phosphorylation of STAT3 (Fig. 3A and B), which indicated that the leptin-induced activation of STAT3 was independent of PI3K and MEK.

To elucidate whether attenuating the Akt and ERK pathways accounted for the upregulation of GRP78 by leptin, SH-SY5Y-ObRb cells were pretreated with LY294002 (5 μM) and PD98059 (10 μM) for 30 min, followed by the leptin treatment (0.5 μg/ml, 24 h). GRP78 levels were analyzed using Western blotting. The PI3K inhibitor hindered the effects of leptin on GRP78 induction (Fig. 3C and D), whereas the MEK inhibitor did not. These results suggested that the leptin-induced expression of GRP78 was dependent on the PI3K/Akt cascade.

### Leptin induced S6K phosphorylation through the PI3K/Akt pathway in SH-SY5Y-ObRb cells

**Figure 4:**
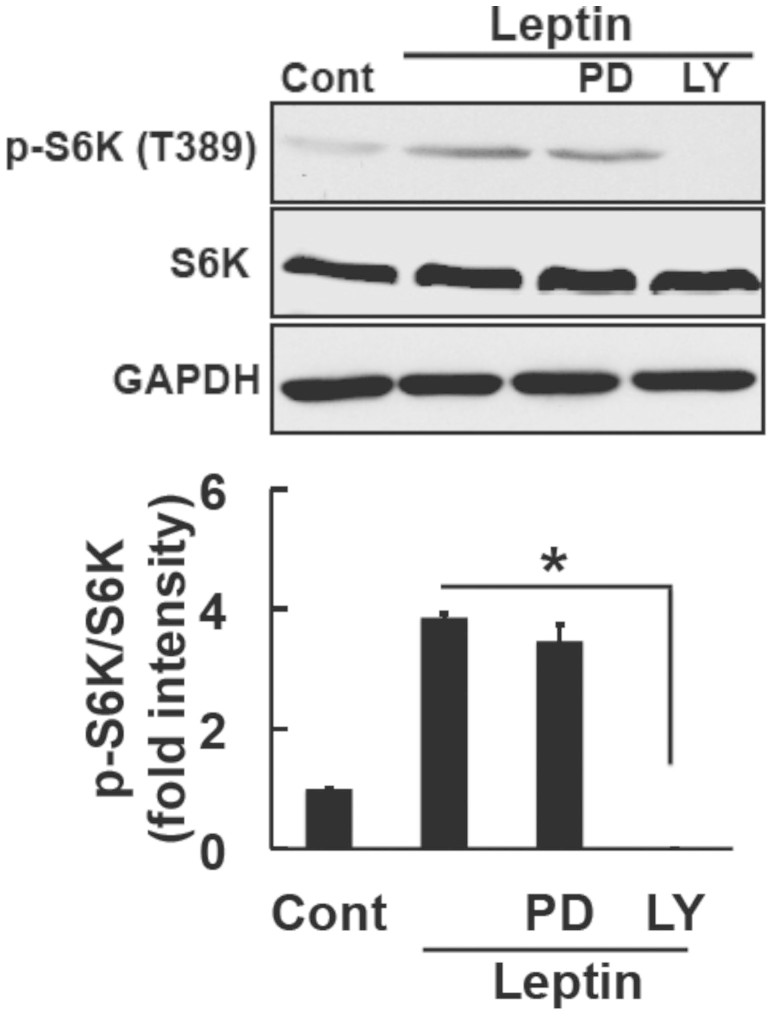
Leptin induced the phosphorylation of S6K through the PI3K/Akt pathway in SH-SY5Y-ObRb cells. SH-SY5Y-ObRb cells were pretreated with PD98059 (PD; 10 μM) or LY294002 (LY; 5 μM) for 30 min followed by leptin (0.5 μg/ml, 30 min). Western blotting analysis was performed using specific antibodies for phospho-S6K (Thr389), S6K, and GAPDH. Densitometric analysis of phospho-S6K (Thr389) using image analyzing software. The leptin-induced phosphorylation of S6K was blocked by LY294002. Data are expressed as the mean ± S.E. of 3 independent experiments (n = 3). * *P* < 0.05.

We subsequently examined one of the downstream regulators of the PI3K/Akt pathway. The activation of Akt is known to participate in important cellular processes such as protein synthesis and cell growth through the mTOR pathway^39^. Therefore, we hypothesized that leptin may activate GRP78 via the Akt/mTOR signaling pathway. Cells were treated with leptin (0.5 μg/ml, 30 min) and p-S6K levels, downstream of mTOR, were analyzed by Western blotting. Consistent with previous findings^40^, we demonstrated that leptin increased p-S6K (Thr389) levels in SH-SY5Y-ObRb cells (Fig. 4). We then examined the effects of LY294002, a PI3K inhibitor, on S6K phosphorylation. SH-SY5Y-ObRb cells were pretreated with LY294002 (5 μM, 30 min) and S6K phosphorylation levels were analyzed by Western blotting. The results obtained showed that the phosphorylation of S6K was blocked by LY294002. Therefore, these results indicated that p-S6K was located downstream of PI3K under leptin-induced signaling in SH-SY5Y-ObRb cells.

### Leptin-induced GRP78 expression was mediated through the mTOR pathway in SH-SY5Y-ObRb cells

**Figure 5:**
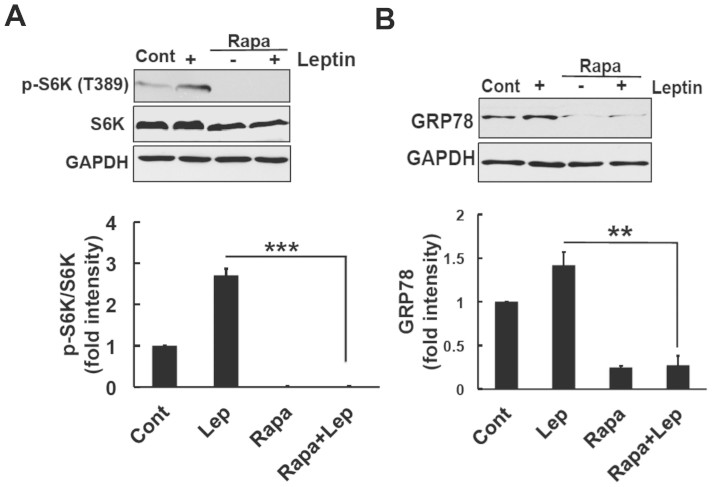
The leptin-induced expression of GRP78 was mediated through the mTOR pathway in SH-SY5Y-ObRb cells. (A). SH-SY5Y-ObRb cells were pretreated with rapamycin (Rapa; 10 nM) prior to the stimulation with leptin (0.5 μg/ml, 30 min). Western blotting analysis was performed using specific antibodies for phospho-S6K (Thr389), S6K, and GAPDH. Densitometric analysis of phospho-S6K (Thr389) using image analyzing software. Rapamycin clearly inhibited leptin-induced S6K phosphorylation. Data are expressed as the mean ± S.E. of 3 independent experiments (n = 3). *** *P* < 0.001. (B). SH-SY5Y-ObRb cells were pretreated with rapamycin (Rapa; 10 nM) and stimulated with leptin (0.5 μg/ml) for 24 h. Western blotting analysis was performed using antibodies for GRP78 and GAPDH. Densitometric analysis was performed using image analyzing software. Rapamycin significantly blocked the leptin-induced expression of GRP78. Data are expressed as the mean ± S.E. of 3 independent experiments (n = 3). ** *P* < 0.01.

We used the mTOR-specific inhibitor, rapamycin to further confirm our results that the leptin-induced expression of GRP78 was dependent on the mTOR pathway. We investigated whether rapamycin could inhibit the phosphorylation of S6K. SH-SY5Y-ObRb cells were pretreated with rapamycin (10 nM) for 30 min followed by leptin (0.5 μg/ml). After 30 min, the phosphorylation levels of S6K were analyzed using Western blotting. We found that rapamycin (10 nM) clearly inhibited the leptin-induced phosphorylation of S6K (Fig. 5A).

We then validated the contribution of the mTOR pathway to the expression of GRP78. Cells were treated with rapamycin (10 nM) and leptin (0.5 μg/ml) for 24 h. Protein was isolated and analyzed by Western blotting. The results showed that rapamycin significantly blocked the leptin-induced expression of GRP78 (Fig. 5B). Therefore, these results suggested that leptin may activate the mTOR pathway, leading to a significant increase in GRP78 levels.

Critical roles of mTOR on leptin signaling have been documented in hypothalamus^41^,^42^. We thus tested the possible effect of rapamycin on leptin signal in our experimental condition. SH-SY5Y-ObRb cells were pretreated with rapamycin (10 nM) for 24 h followed by leptin (0.5 μg/ml, 30 min). As a result, treatment with rapamycin significantly reduced the phosphorylation of STAT3 (Supplementary Fig. 3).

### Involvement of the PI3K/mTOR pathway in the leptin-induced proliferation of SH-SY5Y-ObRb cells

**Figure 6:**
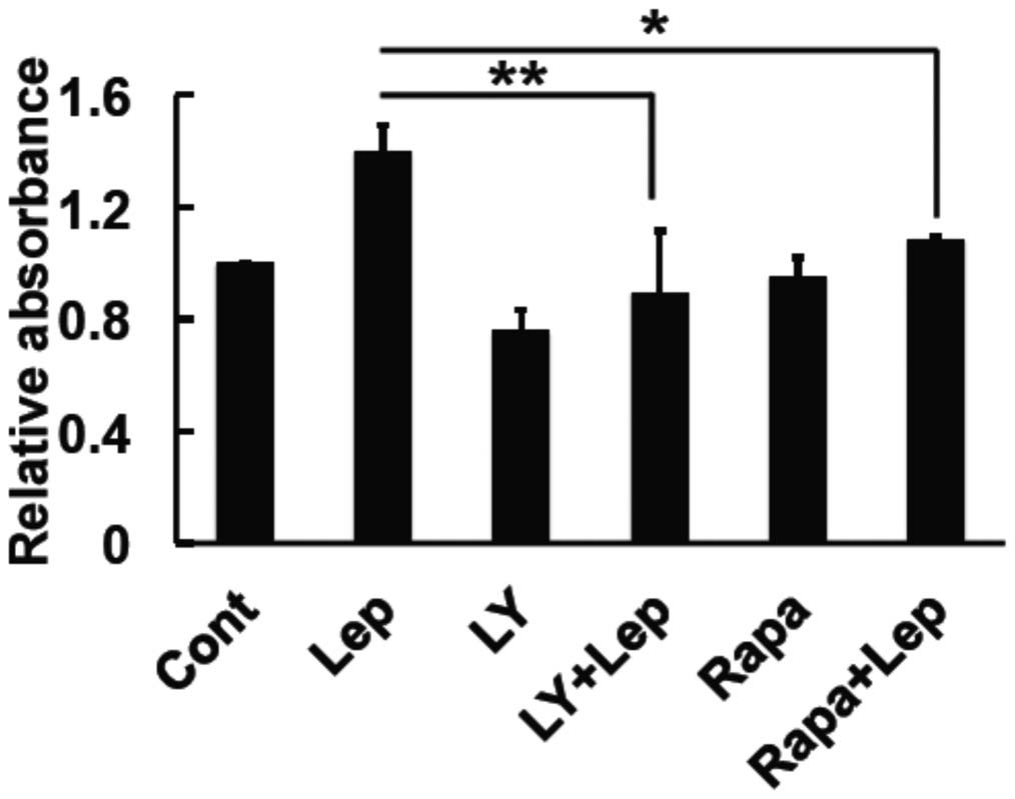
Involvement of the PI3K/mTOR pathway on the leptin-induced proliferation of SH-SY5Y-ObRb cells. SH-SY5Y-ObRb cells were pretreated with LY294002 (LY; 5 μM) or rapamycin (Rapa; 10 nM) for 30 min and then stimulated with leptin (0.5 μg/ml) for 24 h. Cells were incubated with the WST-1 reagent for 4 h and then analyzed for optical density. LY294002 and rapamycin reduced leptin-induced proliferation. Data are expressed as the mean ± S.E. of 4 independent experiments (n = 4). * *P* < 0.05, ** *P* < 0.01.

We demonstrated that the PI3K-mTOR pathway played an important role in the induction of GRP78. We performed a cell proliferation assay to further determine the physiological consequences of the effects of leptin. We investigated the effects of the PI3K inhibitor, LY294002 and mTOR inhibitor, rapamycin on the leptin-induced proliferation of SH-SY5Y-ObRb cells. Forty-eight hours after seeding, cells were washed with serum-free medium and stimulated with LY294002 (5 μM) or rapamycin (10 nM) for 30 min followed by leptin. Twenty-four hours after the treatment, cell growth was measured using the WST-1 assay. As shown in Figure 6, both LY294002 and rapamycin reduced leptin-induced proliferation. These results indicated that activation of the Akt/mTOR pathway mediated the cell proliferative effects of leptin in SH-SY5Y-ObRb cells.

### Leptin protected against ER stress-induced cell death in SH-SY5Y-ObRb cells

**Figure 7:**
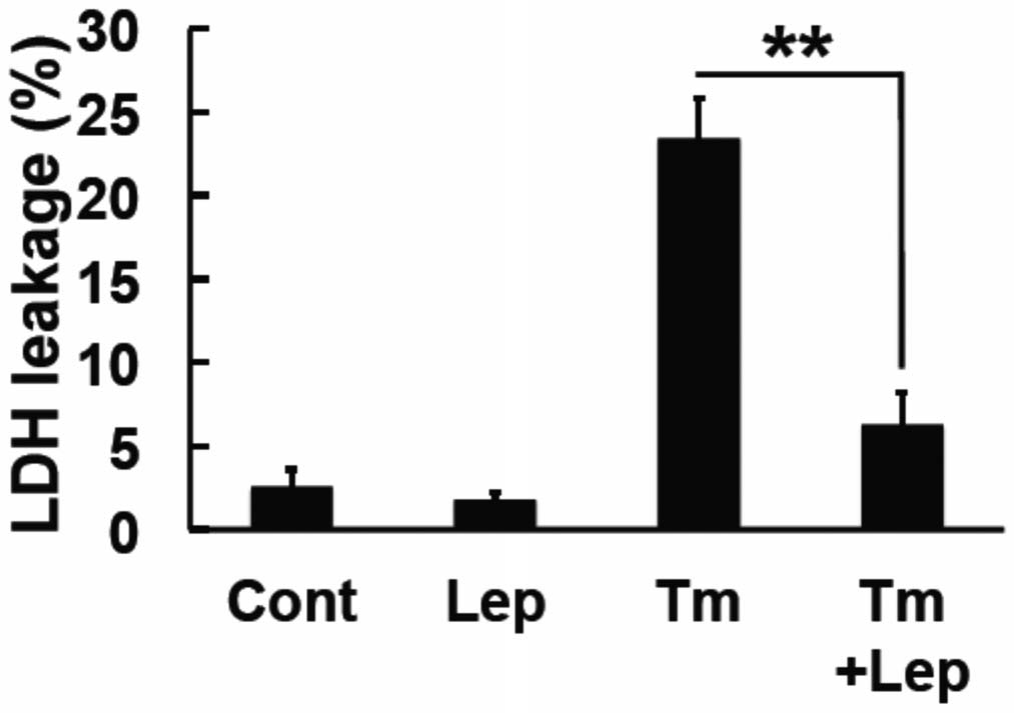
Leptin ameliorated ER stress-induced cell death in SH-SY5Y-ObRb cells. Cells were pretreated with leptin (0.5 μg/ml) for 8 h and then with tunicamycin (Tm, 0.3 μg/ml) for another 48 h in serum-free medium. Lactate dehydrogenase (LDH) activity was measured as the indicator of cytotoxicity. Data are expressed as the mean ± S.E. of 4 independent experiments (n = 4). ** *P* < 0.01.

To evaluate the physiological outcome of leptin against ER stress, we examined its effects on ER stress-induced cell death. Cells were pre-treated with leptin (0.5 μg/ml, 8 h) followed by ER stress inducers (tunicamycin, 0.3 μg/ml, 48 h). Cell death was then measured using the LDH assay. The treatment with tunicamycin caused cell death and this was significantly attenuated by leptin (Fig. 7). Therefore, these results suggested that leptin may be able to attenuate ER stress-induced cell death.

## Discussion

Perturbations in endoplasmic reticulum (ER) homeostasis are known to cause ER stress, which induces cells to trigger the unfolded protein response (UPR). ER stress has been shown to increase with obesity^22^. We and other groups demonstrated that ER stress was involved in the development of leptin resistance^21^,^23^,^24^. In the present study, we found that leptin itself could induce the UPR in SH-SY5Y-ObRb cells (Fig. 2A).

In the present study, we found that leptin can induce GRP78 induction in neuronal cells. The ER stress-induced expression of GRP78 was previously shown to be dependent on the IRE1-XBP1 signaling pathway^32^,^33^. However, we could not detect increase in IRE1 phosphorylation nor XBP1 splicing in leptin-treated cells (Fig. 2C and D). Therefore, leptin-induced GRP78 induction was not mediated through IRE1-XBP1 pathway. Instead of the IRE1-XBP1, we demonstrated that the leptin-induced expression of GRP78 was mediated through the PI3K-mTOR pathway, which was confirmed by the inhibition of leptin-induced GRP78 expression by the PI3K inhibitor, LY294002 (Fig. 3C and D), and mTOR inhibitor, rapamycin (Fig. 5B).

We showed that the PI3K inhibitor significantly inhibited the expression of GRP78 (Fig. 3C and D). In contrast, LY294002 did not inhibit STAT3 phosphorylation (Fig. 3A and B). These results indicated that the JAK2-STAT3 pathway may not be involved in the expression of GRP78. However, we cannot conclude that the leptin-induced expression of GRP78 was not mediated through the JAK2-STAT3 cascade. Further studies using siRNA or STAT3 inhibitors are needed. To the best of our knowledge, we can assume that the PI3K pathway is involved in the induction of GRP78 expression. Leptin signaling and mTOR activity reciprocally function in modulating food intake in hypothalamus^41^,^43^. In the present study, we showed that leptin-induced induction of GRP78 was dependent on PI3K/Akt/mTOR. According to these, we test the possible effect of rapamycin, on leptin signal. Interestingly, treatment with rapamycin reduced basal level of S6K as well as GRP78 levels (Fig. 5A and B). The reasons for these observations are currently unknown but it may be possible that mTOR pathway is involved in GRP78 induction at the basal state. In addition, we found that 24 h-treatment with rapamycin significantly attenuated leptin-induced STAT3 phosphorylation (Supplementary Fig. 3). This result suggested that the rapamycin-mediated reduction in GRP78 expression may result in insensitive to leptin's action. Future study is needed for better understanding the link between GRP78 and leptin signal.

The physiological role of leptin by inducing the expression of GRP78 remains unknown. GRP78 is a key chaperone protein that controls ER homeostasis by enhancing the folding and assembly of proteins^44^,^45^. ER stress was shown to be increased in obesity^46^,^47^,^48^. Previously, we and other groups reported that ER stress caused leptin resistance^21^,^23^,^24^. Therefore, ER stress may be involved in the development of obesity. On the other hand, in the present study, we showed that leptin itself could induce GRP78 but not CHOP in neuronal cells. Based on these observations, we speculated that the physiological role of leptin-induced GRP78 expression is as follows: In the early phase of a stressed condition when leptin resistance has not yet developed, leptin itself may protect against ER stress by inducing the expression of GRP78. Indeed, we found that leptin can protect against ER stress-induced cell death (Fig. 7). On the other hand, resistance may develop when ER stress is severe and prolonged, which leptin cannot afford^21^,^23^. From these observations we would like to propose a novel function of leptin; i.e., by inducing GRP78 expression, leptin may resist against ER stress. Of note, leptin and insulin share the same PI3K/Akt/mTOR pathway in the induction of GRP78. Interestingly, several reports suggested that insulin could enhance GRP78 expression and block the cleavage of PARP and caspase-3 activity by ER stress thereby protect cells from ER stress-induced apoptosis^49^,^50^. Therefore, GRP78 induction mediated through PI3K/Akt/mTOR pathway may be responsible for the protection against ER stress.

The UPR is classically linked to protein-folding stress under both physiological and pathological conditions. Increasing evidence has been reported for the important role of the UPR in physiological responses^51^. For example, the UPR was shown to be involved in regulating innate immunity^52^, energy^53^ and lipid metabolism^54^, and bone formation^55^. The results of the present study suggested that leptin may protect against stress by upregulating the expression of GRP78. Our results may be an example of the physiological function of the UPR activated by leptin.

Besides its well-known role in the regulation of satiety, leptin has been shown to activate a multi-functional signaling pathway involved in proliferation and cancer development^56^. Furthermore, molecular chaperones have been reported to play an important role in tumor growth and survival^57^,^58^.

In conclusion, this is the first study to show that leptin activated the UPR by specifically increasing the expression of GRP78 in a human neuroblastoma cell model. Moreover, the results in this study provide an insight into the novel physiological role of leptin against ER stress-induced cell death.

## Methods

### Material

Tunicamycin (Tm) and thapsigargin (Tg) were obtained from Wako Pure Chemical Ltd. (Japan). LY294002 was purchased from Sigma (MO). PD98059 was provided by Research Biochemical International (MA). Leptin was obtained from Enzo Life Science (NY). Rapamycin was purchased from Santa Cruz (CA).

### Cell culture

Human neuroblastoma (SH-SY5Y) stably transfected with Ob-Rb long form of leptin receptor (SH-SY5Y-ObRb)^30^ were maintained in Dulbecco's modified Eagle's medium supplemented with 10% (v/v) heat-inactivated fetal calf serum at 37°C in humidified 5% CO_2_/95% air. All experiments were performed in Dulbecco's modified Eagle's medium.

### Reverse Transcription-Polymerase Chain Reaction (RT-PCR)

Total RNA was extracted from fresh cells using TriPure Isolation Reagent (Roche). cDNA was reverse transcribed from 2 μg of total RNA by using 25 U of Supercript Reverse Transcriptase (ReverTra Ace) and 0.25 μg of Oligo(dt) 12–18 primer (Life Technology) in a 20-μl reaction mixture containing buffer, 1 mM dNTP mix, 10 mM DTT and 40 U of RNase inhibitor. The total RNA and Oligo(dt) 12–18 primer were incubated at 70°C for 10 min before RT. After incubation for 1.5 h at 46°C, the RT reaction was terminated by denaturing the reverse transcriptase for 5 min at 100°C. Regarding PCR amplification, 1.2 μl of cDNA was added to 10.8 μl of a reaction mix containging 0.2 μM of each primer, 0.2 μM of dNTP mix, 0.6 U of Taq polymerase and reaction buffer. PCR was performed in a DNA Thermal Cycler (MJ Research, PTC-220). The following primers were used: XBP-1; upstream, 5′-CAG CAC TCA GAC TAC GTG CA-3′, and downstream, 5′-CAG AGG TGC ACG TAG TCT GA-3′. GAPDH; upstream, 5′-AAA CCC ATC ACC ATC TTC CAG-3′, and downstream 5′-AGG GGC CAT CCA CAG TCT TCT-3′ was used as an internal control. The PCR products (10 μl) were resolved by electrophoresis in an 8% polyacrylamide gel in TBE buffer. The gel was stained with ethidium bromide and photographed under ultraviolet light.

### Western blotting analysis

Western blotting was performed as described previously^25^,^30^. Cells were washed with ice-cold phosphate-buffered saline and lysed in a buffer containing 10 mM HEPES-NaOH, pH 7.5, 150 mM NaCl, 1 mM EGTA, 1 mM Na_3_VO_4_, 10 mM NaF, 10 μg/ml aprotinin, 10 μg/ml leupeptin, 1 mM phenylmethylsulfonyl fluoride, and 1% Nonidet P-40 for 20 min. The lysates were centrifuged at 15,000 rpm for 20 min at 4°C, and supernatants were collected. The samples were boiled with Laemmli buffer for 3 min, fractionated by SDS-polyacrylamide gel electrophoresis, and transferred at 4°C to nitrocellulose membranes. The membranes were incubated with anti-phospho-Akt (Thr308: Cell Signaling; diluted to 1:1,000), anti-phospho-ERK (Thr202/Tyr204: Cell Signaling; diluted to 1:1,000), anti-CHOP (Santa Cruz; diluted to 1:500), Anti-phospho-IRE1α (phospho S724: Abcam; diluted to 1:1,000), anti-phospho-STAT3 (Tyr705, diluted to 1:1,000; Cell Signaling), anti-KDEL (Enzo Life Sciences; diluted to 1:1,000), anti-phospho-S6K (Thr389: Cell Signaling; diluted 1:1,000), anti-GAPDH (Millipore; diluted to 1: 1,000), anti-ERK1/2 (StressMarq; diluted to 1:5,000), anti-AKT (cell signaling; diluted to 1:1000), anti-STAT3 (Cell Signaling; diluted to 1:1,000), anti-IRE1α (Cell Signaling; diluted to 1:1,000), anti-S6K (Cell Signaling; diluted to 1:1,000), anti-phospho-eIF2α (Cell Signaling; diluted to 1:1,000), anti-ATF6 (Santa Cruz; diluted to 1:500), anti-eIF2α (Cell Signaling; diluted to 1:1,000) antibodies followed by incubation with anti-horseradish peroxidase-linked antibody. Peroxidase was detected by chemiluminescence using an enhanced chemiluminescence using an ECL system (Amersham).

### Lactate dehydrogenase leakage assay (LDH assay)

The viability of cells was estimated by the lactate dehydrogenase (LDH) leakage method using a cytotoxicity detection kit (Roche Molecular Biochemicals, Indianapolis, IN, USA) according to the manufacturer's directions. LDH activity was measured at the optimal density 492 nm.

### WST-1 assay

Cell viability was measured using WST-1 reagent (Roche) according to the manufacturer's directions. Twenty-four hours after the stimulation in serum-free medium, the cells were incubated with WST-1 reagent for 4 h and then analyzed for optical density (440–600 nm).

### Statistics

A one-way analysis of variance analysis was used with Bonferroni's post hoc analysis for comparison between multiple groups. A student's *t*-test was used for comparison between two groups. Significance was defined as a *P* value.

## Author Contributions

M.T. conceived the hypothesis, designed and performed experiments, analyzed the data and wrote the manuscript. T.H. conceived the hypothesis, designed and assisted experiments, analyzed the data and wrote the manuscript. M.Y. assisted experiments. K.O. conceived the hypothesis, analyzed the data.

## Supplementary Material

Supplementary Information Supplementary Figures
