# Supplementary material for: Leptin induced GRP78 expression through the PI3K-mTOR pathway in neuronal cells
Source: Sci Rep. 2014 Nov 18;4:7096. doi: 10.1038/srep07096 (PMC4235288; doi:10.1038/srep07096)
Supplement: Supplementary Information — Supplementary Figures [file srep07096-s1.pdf]

Supplementary Information

Leptin induced GRP78 expression through the PI3K-mTOR pathway in neuronal cells

Mina Thon[1], Toru Hosoi[1]*, Michiko Yoshii[1], Koichiro Ozawa[1]*

[1]Department of Pharmacotherapy, Graduate School of Biomedical and Health Sciences, Hiroshima University, 1-2-3 Kasumi, Minami-ku, Hiroshima 734-8551, Japan

*Correspondence to: Koichiro Ozawa (e-mail: ozawak@hiroshima-u.ac.jp) or Toru Hosoi (e-mail: toruh@hiroshima-u.ac.jp), Department of Pharmacotherapy, Graduate School of Biomedical and Health Sciences, Hiroshima University, 1-2-3 Kasumi, Minami-ku, Hiroshima 734-8551, Japan. TEL: +81-82-257-5338

Supplementary information includes:

Supplementary Figures S1 to S4

Supplementary Figure 1.

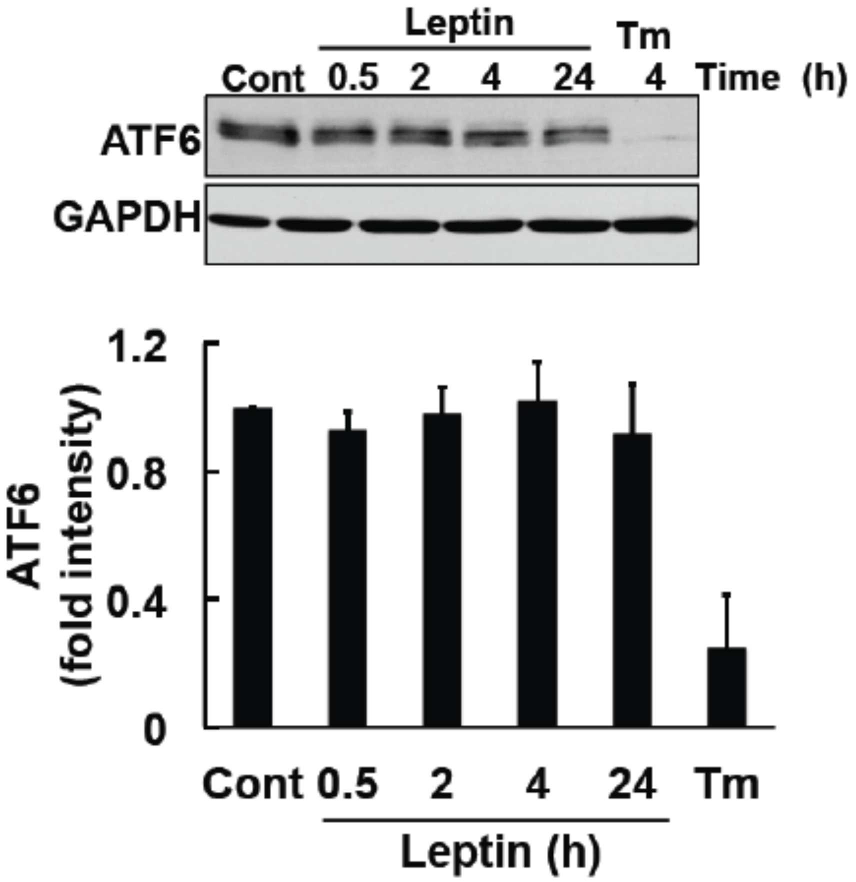

Supplementary Figure 1. Leptin did not induce ATF6 processing. SH-SY5Y-ObRb cells were treated with leptin (0.5 µg/ml) for 0.5, 2, 4, and 24 h. Tunicamycin (Tm) (1 µg/ml, 4 h) was used as the positive control. Densitometric analysis of ATF6 levels using image analyzing software. Typical data of 3 independent experiments were shown.

Supplementary Figure 2.

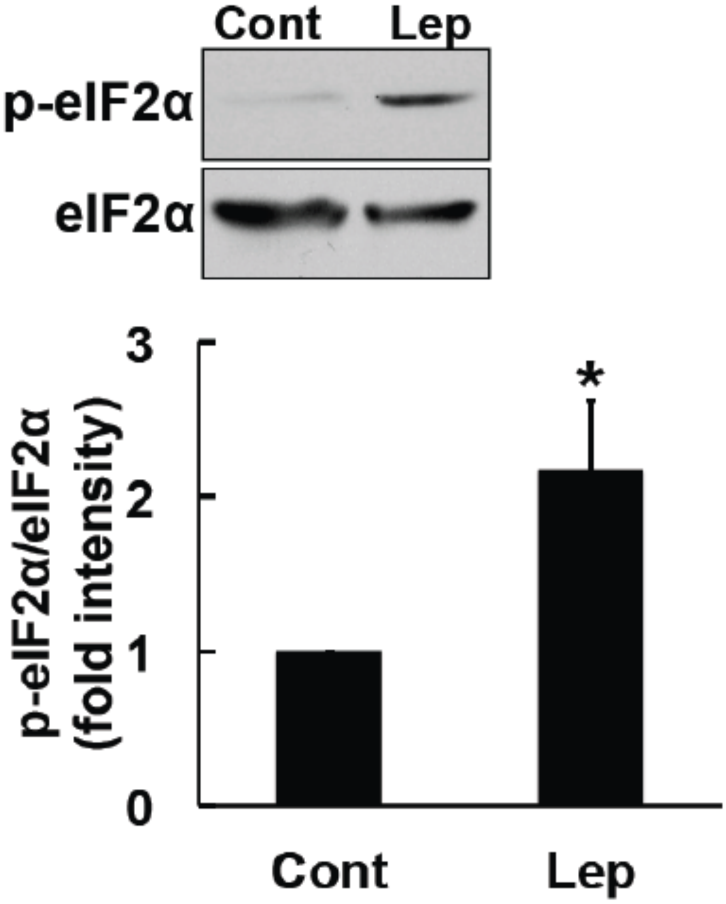

Supplementary Figure 2. Leptin increased the phosphorylation of eIF2α. SH-SY5Y-ObRb cells were treated with leptin (0.5 μg/ml) for 4 h. Densitometric analysis of p-eIF2α levels using image analyzing software. Data are expressed as the mean ± S.E. of 4 independent experiments (n = 4). * $P < 0.05$.

Supplementary Figure 3.

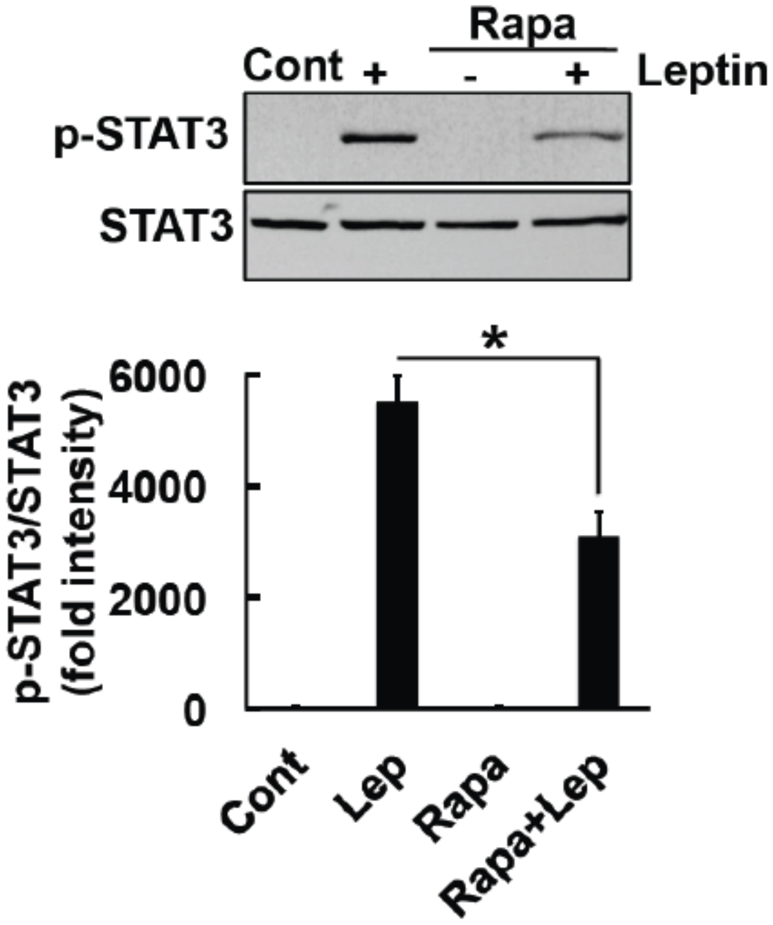

Supplementary Figure 3. Rapamycin inhibited leptin signal in SH-SY5Y-ObRb cells. SH-SY5Y-ObRb cells were pretreated with rapamycin (10 nM) for 24 h followed by leptin (0.5 μg/ml, 30 min). Western blotting analysis was performed using specific antibodies for phospho-STAT3 (Tyr705) and STAT3. Densitometric analysis of phospho-STAT3 (Tyr705) levels using image analyzing software. The leptin-induced phosphorylation of STAT3 was inhibited by rapamycin. Data are expressed as the mean ± S.E. of 4 independent experiments (n = 4). * $P < 0.05$.

Supplementary Figure 4.

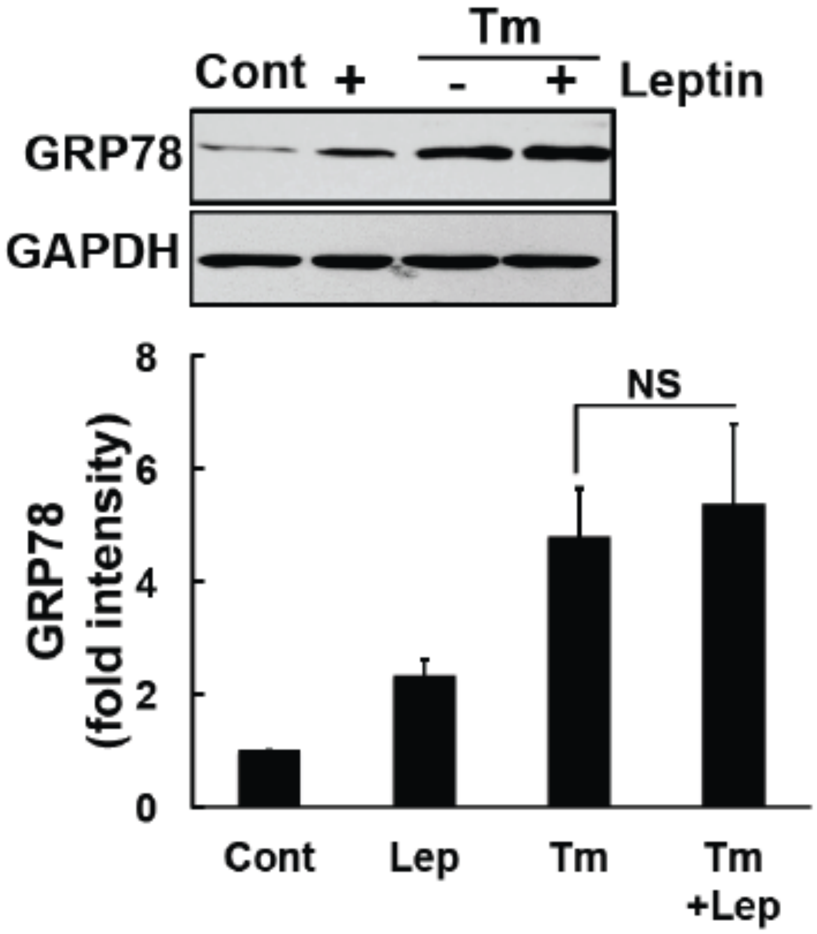

Supplementary Figure 4. GRP78 levels would not be increased in ER stressed model. SH-SY5Y-ObRb cells were pretreated with Tm (0.05 μg/ml) for 4 h followed by leptin (0.5 μg/ml, 24 h). Western blotting analysis was performed using antibodies for GRP78 and GAPDH. Densitometric analysis of GRP78 using image analyzing software. Data are expressed as the mean ± S.E. of 3 independent experiments (n = 3).
